# Presence of sarcopenia does not affect the clinical results of balloon kyphoplasty for acute osteoporotic vertebral fracture

**DOI:** 10.1038/s41598-020-80129-z

**Published:** 2021-01-08

**Authors:** Shoichiro Ohyama, Masatoshi Hoshino, Shinji Takahashi, Yusuke Hori, Hiroyuki Yasuda, Hidetomi Terai, Kazunori Hayashi, Tadao Tsujio, Hiroshi Kono, Akinobu Suzuki, Koji Tamai, Hiromitsu Toyoda, Sho Dohzono, Hiroaki Nakamura

**Affiliations:** 1 Department of Orthopaedic Surgery, Osaka City University Graduate School of Medicine, 1-4-3 Asahi-machi, Abeno-ku, Osaka, 545-8585 Japan; 2 Department of Orthopaedic Surgery, Osaka General Hospital of West Japan Railway Company, Osaka, Japan; 3 Department of Orthopaedic Surgery, Shiraniwa Hospital, Nara, Japan; 4 Department of Orthopaedic Surgery, Ishikiri Seiki Hospital, Osaka, Japan; 5 Department of Orthopaedic Surgery, Yodogawa Christian Hospital, Osaka, Japan

**Keywords:** Outcomes research, Fracture repair, Geriatrics, Quality of life, Skeletal muscle, Bone, Trauma

## Abstract

Sarcopenia has been associated with poor clinical outcomes in several diseases. Herein, the clinical results of balloon kyphoplasty (BKP) for acute osteoporotic vertebral fracture (OVF) treatment were assessed and compared between sarcopenia and non-sarcopenia patients. Sixty patients who underwent BKP for treatment of acute OVF with poor prognostic factors between April 2016 and September 2017 and were assessed for sarcopenia were enrolled. Clinical results (back pain on visual analogue scale [VAS]; short-form [SF] 36; vertebral deformity; activities of daily living levels; and incidence of adjacent vertebral fractures) were compared between the two groups at 6 months post-BKP. Data analysis revealed that back pain on VAS, SF-36 scores, and vertebral deformity improved from baseline to 6 months after BKP. Thirty-nine patients (65.0%) were diagnosed with sarcopenia and demonstrated a lower body mass index (21.2 vs. 23.3 kg/m^2^, p = 0.02), skeletal muscle mass index (5.32 vs. 6.55 kg/m^2^, p < 0.01), hand-grip strength (14.7 vs. 19.2 kg, p = 0.01), and bone mineral density of the femoral neck (0.57 vs. 0.76 g/cm^2^, p < 0.01) than those of patients without sarcopenia. However, no significant differences were observed in the clinical results between these groups. Therefore, BKP’s clinical results for the treatment of acute OVF are not associated with sarcopenia.

## Introduction

Sarcopenia is described as an age-related decline in skeletal muscle mass as well as muscle function, and has recently been attracting a great deal of attention^1,2^. The prevalence of sarcopenia rises with the increasing age of the population, therefore, sarcopenia has become increasingly common in the elderly, particularly in the super-aged society we are currently facing^3^. It has been reported that sarcopenia is related to the high mortality observed in community-dwelling elderly people^4^. With respect to cancer, cardiovascular disease, and chronic kidney disease, sarcopenia has been reported to be a potential risk for poor clinical outcomes^5–8^. Patients with sarcopenia were reported to have a high incidence of osteoporosis^3,9^, and thus have a high incidence of osteoporotic vertebral fractures (OVFs)^10,11^.

OVF have been reported to be the most frequently occurring fragile fracture among the elderly^12^, with a 50% prevalence in Japanese females over the age of 80^13^. Most patients with acute OVF responded well to conservative treatment^14^; however, some patients with acute OVF reported poor outcomes such as persistent back pain and a decline in the level of activities of daily living (ADL) due to the delayed union of OVF^15,16^. MRI findings (T2 weighted images) of the acute OVF predicted the potential risk of delayed union, and they were called poor prognostic factors^17,18^. Balloon kyphoplasty (BKP) for acute OVF with poor prognostic factors was considered superior to conservative management^19^; however, no reports indicate that BKP intervention would also be effective for patients with sarcopenia. We hypothesized that the clinical results of BKP for acute OVF with poor prognostic factors differ between patients with and without sarcopenia, and like various other diseases, patients with sarcopenia would have poorer outcomes when compared to patients without sarcopenia.

This study aimed to compare the clinical results of BKP for acute OVF with poor prognostic factors between patients with and without sarcopenia.

## Methods

### Ethical approval

This was a multicenter, prospective BKP intervention study for acute OVFs with poor prognostic factors detected with MRI^17,18^. It was carried out among 10 institutions within the Osaka area of Japan between April 2015 and September 2017. This study protocol was approved by the Institutional Review Board of Osaka City university (No. 3174). All methods were performed in accordance with the Declaration of Helsinki and the Ethical Guidelines for Medical and Health Research Involving Human Subjects in Japan.

### Patients

Elderly patients (aged ≥ 65 years) who had developed an acute OVF with poor prognostic factors (a high-intensity or diffuse low-intensity area in fractured vertebrae on T2 weighted MRI) within the last two months were enrolled for the study. The MRI findings were previously reported to be associated with delayed union, reduced ADLs, and intractable back pain at 6 months^17,18^.

**Figure 1:**
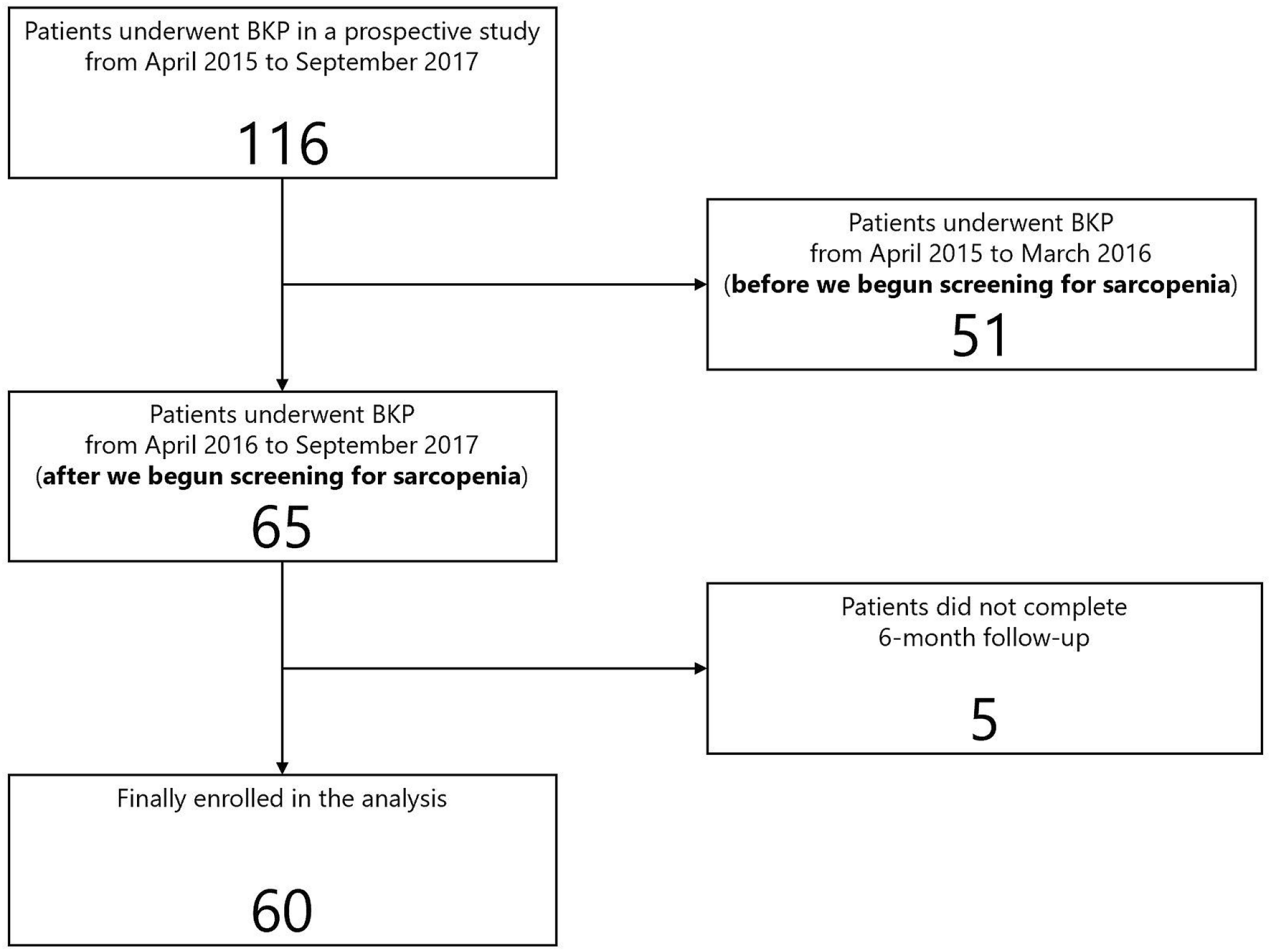
Flow chart for this study.

On the initial visit, if the patient had acute back pain; a deformed vertebral body on radiographs, and abnormal intensity within the vertebral bodies on MRI, it was diagnosed as a new OVF. These fractures were caused by low-energy injury mechanisms, such as spontaneous fractures and falls while standing. Patients with neurological deficits, pathological fractures, and suspected underlying malignant diseases were excluded. The enrolled patients had back pain measuring 40 mm or more on the Visual Analogue Scale (VAS). All patients provided written informed consent before enrollment, and a total of 116 patients were enrolled for BKP. From April 2016, we began screening for sarcopenia using the AWGS 2014 criteria. A total of 60 patients who were able to both undergo screening for sarcopenia within two months after BKP and complete the 6-month follow-up were enrolled in this analysis (Fig. 1).

### Clinical results

**Figure 2:**
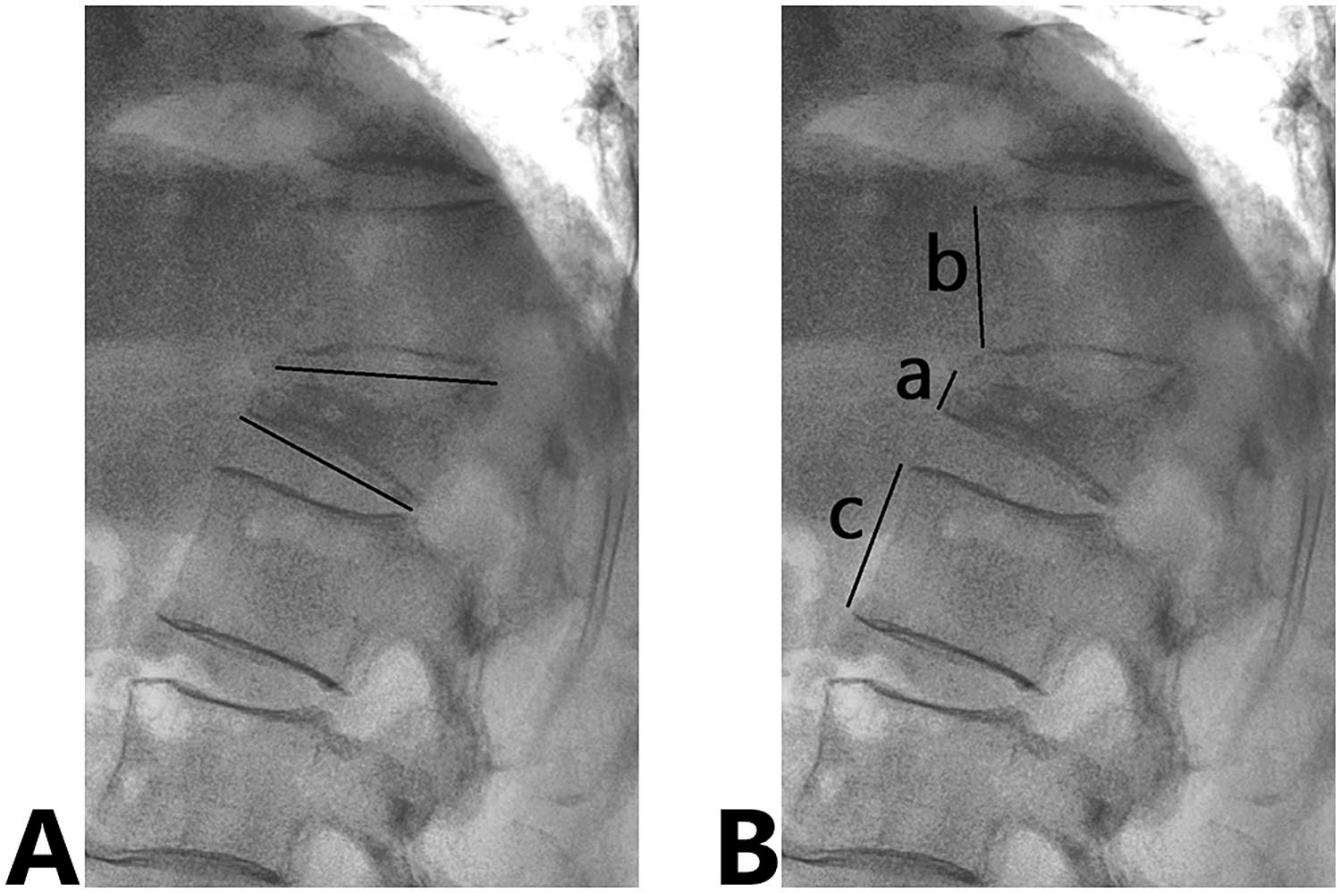
Assessment of the vertebral deformity. (**A**) The vertebral wedge angle was measured from the lateral view of radiograph in weight-bearing position. (**B**) The percentage vertebral body height was calculated by the following formula: (2 a/[b + c] × 100.

**Figure 3:**
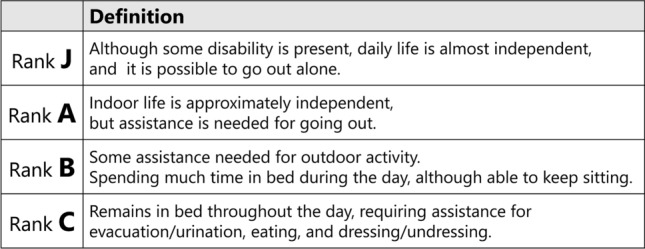
Criteria for evaluating the degree of independence (severity of bed-ridden state) during daily living for disabled elderly people. In brief, Rank J indicate independent, Rank A indicates requires assistance to leave home, Rank B indicates nearly-bed ridden, and Rank C indicates completely-bed-ridden.

We assessed back pain on the VAS, short-form 36 subscales (SF-36), and vertebral body deformity (vertebral body wedge angle and percentage (%) vertebral body height) both at the initial visit (baseline) and 6 months after BKP. The vertebral wedge angle was measured from the lateral view of radiographs in a weight-bearing position. The vertebral body height was calculated using the formula: (2 anterior vertebral height of affected vertebra/sum of anterior vertebral height of upper and lower vertebra) × 100 (Fig. 2). Patients’ ADL before OVF and 6 months after BKP were evaluated using the criteria proposed by the Long-term Care Insurance System in the Japanese Health and Welfare Ministry (Fig. 3). The incidence of radiologic adjacent vertebral fractures (AVF), defined as a new OVF above or below the affected vertebra, was observed via computed tomography (CT) 6 months after the BKP, regardless of whether symptoms are shown.

### Diagnosis of sarcopenia (AWGS 2014 criteria)

We diagnosed the patients with sarcopenia according to the diagnostic algorithm of AWGS 2014^20^ within 2 months after the BKP. In the AWGS 2014 algorithm, elderly people with low muscle mass, in addition to low hand-grip strength and/or slow walking speed, are diagnosed with sarcopenia.

### Muscle mass

Muscle mass was measured using a bioelectrical impedance analysis (BIA) machine (MC780A, TANITA, Japan). Appendicular skeletal muscle mass (ASM) was calculated as the sum of skeletal muscle mass of the arms and legs. Skeletal muscle mass index (SMI) was defined as ASM divided by height in meters squared (ASM/height^2^)^1^. SMI of < 7.0 kg/m^2^ in males and < 5.7 kg/m^2^ in females was defined as a low muscle mass.

### Hand-grip strength

Hand-grip strength was measured using a hand-held dynamometer (T.K.K.5401, TAKEI, Japan). Two trials were carried out for each hand; the highest value was recorded for the assessment^20^. Hand-grip strength of < 26 kg in males and < 18 kg in females was defined as a low hand-grip strength.

### Usual gait speed

Patients were instructed to walk at their usual pace over an 8-m course, stopping just after the finish line. Excluding the first and last meter, the time taken to walk through the central 6 m was measured. Usual gait speed (m/s) was calculated using the time taken to complete the 6-m walk^21^. Usual gait speed of < 0.8 m/s was defined as a slow walking speed.

### Statistical analysis

For the analyses, patients were divided into two groups; patients diagnosed with (Sarcopenia group), and without sarcopenia (No Sarcopenia group). The Chi-squared (χ^2^) or Fisher’s exact tests were used for categorical variables, and the t-test was used for continuous variables. When comparing clinical results at 6 months after BKP, the analysis of covariance was used to adjust for baseline values as covariates. Statistical test results were considered significant for values of p < 0.05, and all p-values were two-sided. All statistical analyses were performed using EZR (Saitama Medical Center, Jichi Medical University, Saitama, Japan), a graphical user interface for R software version 3.6.3. (R Core Team (2020). R: A language and environment for statistical computing. R Foundation for Statistical Computing, Vienna, Austria. URL https://www.R-project.org/). More precisely, the program is a modified version of R commander designed to add statistical functions frequently used in biostatistics^22^.

Sample size calculations assumed a difference of 16 mm in the VAS of back pain. Using an estimated standard deviation of 20 mm and accepting a two-sided type I error rate of 5%, we would achieve 80% power to detect a difference (effect size, 0.75) with 25 patients per arm. However, this was a study with two unequal groups. Individual sample sizes in the two groups were calculated 19 and 38 patients (a total of 57 patients). The number of the patients in this study was 60 and it satisfied the sufficient sample size.

## Results

The background data of patients are presented in Table 1. The mean age of patients was 77.8 years, and 78.3% of the patients were women. Eighty-five percent of OVFs were found between T10 and L2. Overall, the clinical results, such as VAS scores for low back pain, SF-36 scores, and vertebral deformity were improved from baseline to 6 months (Table 2). Despite having suffered from an acute OVF with poor prognostic factors, 96.7% of the patients maintained their original ADL levels at 6 months after BKP.

**Table 1:** Background data of the patients with acute OVF with poor prognostic factors.

	Total N = 60
Age, years	77.8 ± 5.8
Female	78.3% (47)
BMI, kg/m^2^	21.9 ± 3.5
Hand-grip strength, kg	16.3 ± 6.9
Gait speed, m/s	0.81 ± 0.28
SMI, kg/m^2^	5.75 ± 0.98
BMD (femoral neck), g/cm^2^	0.64 ± 0.25
**Baseline prevalent fractures**	
0	45.0% (27)
1	31.7% (19)
≥ 2	23.3% (14)
**Level of OVF**	
T10-L2	85.0% (51)
L3-L5	15.0% (9)
**ADL before OVF**	
J	81.7% (49)
A	15.0% (9)
B	3.3% (2)
C	0.0% (0)

Data are presented as mean ± standard deviation.

Student t-test for continuous variables and Chi-squared test for categorical variables were used to compare groups.

*OVF* osteo porotic vertebral fracture, *BMI* body mass index, *SMI* skeletal muscle mass index, *BMD* bone mineral density, *ADL* activity of daily living.

**Table 2:** Comparison of clinical results of patients underwent BKP for acute OVF with poor prognostic factors between baseline and 6 months.

	Baseline	6 months	p
VAS of back pain, mm	73.9 ± 26.8	29.5 ± 25.8	< 0.01
PCS of the SF36	3.20 ± 13.2	25.6 ± 19.3	< 0.01
MCS of the SF36	37.8 ± 11.4	51.5 ± 9.4	< 0.01
Vertebral body wedging angle, °	17.5 ± 7.8	12.4 ± 6.5	< 0.01
% vertebral body height, %	48.8 ± 19.9	65.7 ± 13.1	< 0.01
Radiological AVF, % (n)		40.0% (24)	

	Before OVF	6 months	
**ADL, % (n)**			1.00
J	81.7% (49)	83.3% (50)	
A	15.0% (9)	13.3% (8)	
B	3.3% (2)	3.3% (2)	
C	0.0% (0)	0.0% (0)	
Reduced ADL, % (n)		3.3% (2)	

Data are presented as mean ± standard deviation.

Paired t-test for continuous variables and Chi-squared test for categorical variables were used to compare Baseline and 6 months.

*BKP* balloon kyphoplasty, *OVF* osteoporotic vertebral fracture, *VAS* visual analogue scale, *SF36* short form 36, *PCS* physical component summary, *MCS* mental component summary, *AVF* adjacent vertebral fracture, *ADL* activity of daily living.

### Prevalence of sarcopenia

Of the 60 patients, 39 were diagnosed with sarcopenia using the AWGS 2014 criteria, making the prevalence of sarcopenia in this study group 65.0%.

### Comparison between the sarcopenia and no sarcopenia group

The sarcopenia group was observed to have a lower BMI (21.2 kg/m^2^ vs. 23.3 kg/m^2^, p = 0.02), hand-grip strength (14.7 kg vs. 19.2 kg, p < 0.01), SMI (5.32 kg/m^2^ vs. 6.55 kg/m^2^, p < 0.01), and bone mineral density of the femoral neck (0.57 g/cm^2^ vs. 0.76 g/cm^2^, p < 0.01) than the no sarcopenia group. There was however no significant difference in the age, gender, level of OVF, or ADL levels before OVF observed between the two groups (Table 3).

**Table 3:** Comparison of patients’ background data between sarcopenia and no sarcopenia group.

	Sarcopenia N = 39	No sarcopenia N = 21	p
Age, years	78.4 ± 5.6	76.9 ± 5.9	0.34
Female, % (n)	76.2% (31)	79.5% (16)	0.76
BMI, kg/m^2^	21.2 ± 2.8	23.3 ± 4.1	0.02
Hand-grip strength, kg	14.7 ± 5.4	19.2 ± 8.4	0.01
Gait speed, m/s	0.78 ± 0.27	0.88 ± 0.27	0.19
SMI, kg/m^2^	5.32 ± 0.63	6.55 ± 1.04	< 0.01
BMD (femoral neck), g/cm^2^	0.57 ± 0.20	0.76 ± 0.27	< 0.01
**Baseline prevalent fractures**			0.88
0	43.6% (17)	47.6% (10)	
1	30.8% (12)	33.3% (7)	
≥ 2	25.6% (10)	19.0% (4)	
**Level of OVF, % (n)**			0.99
T10-L2	84.6% (33)	85.7% (18)	
L3-L5	15.4% (6)	14.3% (3)	
**ADL before OVF, % (n)**			0.86
J	79.5% (31)	85.7% (18)	
A	15.4% (6)	14.3% (3)	
B	5.1% (2)	0.0% (0)	
C	0.0% (0)	0.0% (0)	

Data are presented as mean ± standard deviation.

Student t-test for continuous variables and Chi-squared test for categorical variables were used to compare groups.

*BMI* body mass index, *SMI* skeletal muscle mass index, *BMD* bone mineral density, *OVF* osteoporotic vertebral fracture, *ADL* activity of daily living.

**Figure 4:**
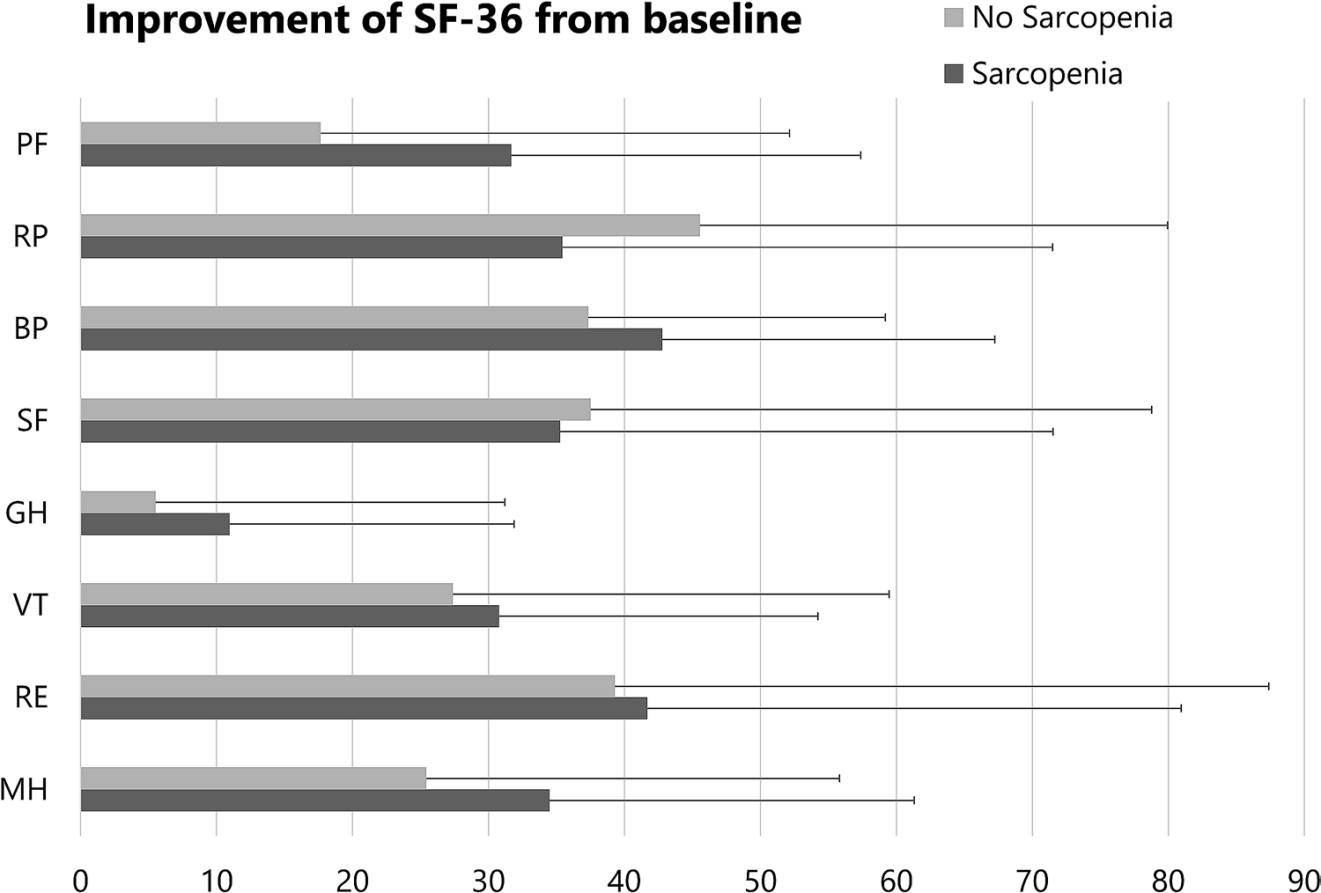
Comparison of the improvement of SF-36 subscale scores from baseline to 6 months after BKP between Sarcopenia group and No sarcopenia group. There was no significant difference in any subscales. *PF* physical functioning, *RP* role physical, *BP* bodily pain, *SF* social functioning, *GH* general health, *VT* vitality, *RE* role emotional, *MH* mental health. n. Bars denote statistically significant differences.

Adjusted by baseline, there was no significant difference in the VAS for low back pain, Physical Component Summary (PCS) of the SF-36, Mental Component Summary (MCS) of the SF-36, and vertebral deformity between the two groups at the 6-months follow-up after BKP (Table 4). Detailed analysis of the SF-36 revealed no significant differences in any subscales (Fig. 4). The incidence of radiological AVF (sarcopenia group: 38.5%, no sarcopenia group: 42.9%) and reduced ADL levels (sarcopenia group: 2.6%, No Sarcopenia group: 4.8%) were also not significantly different between the two groups.

**Table 4:** Comparison of patients’ clinical results between sarcopenia and no sarcopenia group.

	Sarcopenia N = 39	No sarcopenia N = 21	p
**VAS of back pain, mm**			
Baseline	78.4 ± 22.5	65.5 ± 32.0	0.08
6 months	26.1 ± 25.0	36.7 ± 27.4	0.11
**PCS of the SF36**			
Baseline	1.72 ± 12.6	5.77 ± 14.1	0.26
6 months	24.4 ± 18.2	27.7 ± 21.4	0.80
**MCS of the SF36**			
Baseline	37.2 ± 11.5	38.7 ± 11.2	0.63
6 months	52.1 ± 9.8	50.4 ± 8.7	0.44
**Vertebral body wedging angle, °**			
Baseline	17.5 ± 8.2	17.4 ± 7.4	0.96
6 months	12.3 ± 7.1	12.5 ± 5.5	0.51
**% vertebral body height, %**			
Baseline	47.4 ± 21.1	51.1 ± 17.7	0.51
6 months	63.9 ± 14.2	69.0 ± 10.1	0.32
Radiological AVF, % (n)	38.5% (15)	42.9% (9)	0.78
Reduced ADL, % (n)	2.6% (1)	4.8% (1)	1.00

Data are presented as mean ± standard deviation.

Student t-test for continuous variables of Baseline was used to compare groups.

Analysis of covariance for continuous variables of 6 months was used to compare groups adjusted by Baseline.

Chi-squared test for categorical variables was used to compare groups.

*VAS* visual analogue scale, *SF36* short form 36, *PCS* physical component summary, *MCS* mental component summary, *AVF* adjacent vertebral fracture, *ADL* activity of daily living.

## Discussion

This is the first study to compare the clinical results of BKP intervention for an acute OVF with poor prognostic factors between patients with and without sarcopenia. Unlike previous reports on various other diseases, no difference was observed in the clinical results between patients with and without sarcopenia.

The prevalence of sarcopenia in this study population (65.0%) was remarkably higher than that of the community-dwelling elderly population (11.2% between ages 75–79 years and 27.0% above 80 years)^3^, a finding similar to those of previous studies on the relationship between sarcopenia and OVF. It may thus be assumed that the majority of patients receiving treatment for OVF must also have sarcopenia.

Acute OVF induces severe low back pain and restricts ADL in affected patients. The incidence of low back pain was higher in overweight individuals (BMI 25.0–29.9) than in those with a healthy weight (BMI 18.5–24.9). Based on a meta-analysis of cohort studies, Zhang et al. reported that an odds ratio (OR) for the incidence of back pain for overweight versus healthy weight individuals was 1.15 (95% [CI] 1.08–1.21)^23^. It has recently been established that BMI has a causal effect on back pain (OR 1.15 per 1-standard deviation [SD: 4.65 kg/m^2^] increase in BMI [95% [CI] 1.06–1.25]) and chronic back pain (OR: 1.20 per 1 SD increase in BMI [95% [CI] 1.09–1.32])^24^. It is therefore possible that the lower BMI of the patients with sarcopenia may have had a positive effect on the clinical results of the BKP, counteracting the disadvantages of sarcopenia reported in various other diseases.

A decrease in trunk muscle mass and the degeneration of back muscles were reported to be associated with lower back pain^25,26^. However, the diagnostic criteria for sarcopenia did not include the assessment of either trunk muscle mass or back muscle degeneration; rather, it included assessment of the appendicular skeletal muscle mass^20^. Therefore, diagnosis of sarcopenia according to present criteria did not reflect the decline in trunk muscle mass or back muscle degeneration in the patients. This was considered to be another reason as to why patients with sarcopenia did not demonstrate poor clinical results in this study.

Conversely, Iida et al. reported that sarcopenia significantly affected the Barthel indices recorded at the first visit and upon discharge of patients who underwent hospitalization and conservative treatment for acute OVFs. Patients with sarcopenia were significantly more likely to be discharged to a nursing home within a year of being discharged than patients without sarcopenia. Therefore, they concluded that sarcopenia affected the outcomes of conservative therapy for OVF, and suggested that treatment of sarcopenia was necessary to improve clinical outcomes of OVF treatment^27^. Unfortunately, despite the amount of research available in the field, there is no treatment for sarcopenia with strong supporting evidence as yet^28^. It thus remains difficult to treat an acute OVF via conservative treatment that includes an effective treatment for sarcopenia.

Compared to conservative treatment, BKP intervention for acute OVF with poor prognostic factors was reported to be better at preventing the ADL decline observed in patients (BKP; 5.6% vs. Conservative treatment; 25.6%)^19^. Considering the results of prior studies in conjunction with our own, BKP intervention should be preferred over conservative treatment for acute OVF with poor prognostic factors in patients with sarcopenia.

This study has several limitations. Firstly, the assessment of sarcopenia was not performed before carrying out the BKP intervention; therefore, we could not explore the influence of BKP intervention on sarcopenia. Secondly, as not all patients had received whole spine radiographs, we could not measure the spinopelvic parameters of the patients in this study. Previous studies have reported that individuals with sarcopenia have a larger sagittal vertebral axis (SVA) than those without sarcopenia in cases of spinopelvic mismatch^29^; larger SVA has been associated with a poor quality of life and persistent back pain^30^. Although we could not discuss the relationship between sarcopenia and spinopelvic parameters, no significant differences were noted regarding the quality of life and degree of back pain experienced between patients with and without sarcopenia. Lastly, the follow-up duration of this study was relatively short at only 6 months. According to studies with a long-term follow-up duration, sarcopenia was reported to increase the mortality in the elderly^4^. Thus, there is scope for future research, as this suggests that differences between patients with and without sarcopenia will be clearer with studies involving a long-term follow-up.

## Conclusion

This study investigated differences in the clinical results of BKP performed for treatment of acute OVF with poor prognostic factors between patients with and without sarcopenia; no significant differences were revealed. We therefore concluded that the clinical results of BKP for acute OVF are not affected by the presence of sarcopenia.
